# The perceived behavior and barriers of community care professionals in encouraging functional activities of older adults: the development and validation of the MAINtAIN-C questionnaire

**DOI:** 10.1186/s12913-020-05762-w

**Published:** 2020-09-29

**Authors:** Ruth G. M. Vogel, Gerrie J. J. W. Bours, Silke F. Metzelthin, Petra M. G. Erkens, Gerard J. P. van Breukelen, Sandra M. G. Zwakhalen, Erik van Rossum

**Affiliations:** 1 Care and Public Health Research Institute, Department of Health Services Research, Maastricht University, Maastricht, The Netherlands; 2 Living Lab in Ageing and Long-Term Care, Maastricht, The Netherlands; 3 Research Centre for Community Care, Academy of Nursing, Zuyd University of Applied Sciences, Heerlen, The Netherlands; 4 Care and Public Health Research Institute, Department of Methodology and Statistics, and Graduate School of Psychology and Neuroscience, Maastricht University, Maastricht, The Netherlands

**Keywords:** Nurses, Community health, Aged, Functional activities, Questionnaires, Psychometrics

## Abstract

**Background:**

Community care professionals need to encourage older adults in performing functional activities to maintain independence. However, professionals often perform functional activities on behalf of older adults. To change this, insights into the behavior and barriers of professionals in encouraging activities are required. In the current study, the MAINtAIN questionnaire, which was developed for nursing homes, was adopted. The objective was to create a modified version that is suitable for measuring behavior and barriers of community care professionals in encouraging functional activities of clients in the community care setting. The overall aims were to assess the content validity, construct validity, and internal consistency of the modified version.

**Methods:**

Data was collected by qualitative and quantitative methods in two phases. During phase one, the MAINtAIN was assessed on appropriateness and feasibility by community nurses *(N* = 7), and the adapted questionnaire was assessed on content validity by research experts *(N* = 9) and community care professionals *(N* = 18). During phase two, the psychometric properties of the adapted MAINtAIN-C were assessed in community care professionals *(N* = 80). Construct validity was evaluated by an Exploratory Factor Analysis (EFA), and internal consistency was determined by calculating Cronbach’s alpha coefficients.

**Results:**

The formulation, verbs, and wording of the MAINtAIN were adapted; some items were excluded and relevant items were added, resulting in the MAINtAIN-C with two scales, showing good content validity. The Behaviors scale (20 items) measures perceived behavior in encouraging functional activities, expressing good internal consistency (Cronbach’s alpha: .92). The Barriers scale measures barriers in encouraging functional activities related to two dimensions: 1) the clients’ context (7 items), with good internal consistency (.78); and 2) the professional, social, and organizational contexts (21 items), showing good internal consistency (.83).

**Conclusions:**

The MAINtAIN-C seems promising to assess the behavior and barriers of community care professionals in encouraging functional activities. It can be used to display a possible difference between perceived and actual behavior, to develop strategies for removing barriers in encouraging activities to foster behavioral change. The results also provide guidance for further research in a larger sample to obtain more insight into the psychometric properties.

## Background

The current ageing policy in the Netherlands is focused on enabling older adults to maintain independence in daily living and continue living in their homes as long as possible [1]. This implies that more older adults remain in the community to prevent or postpone institutional care [2]. To facilitate the current ageing policy, individuals need to have the ability to perform functional activities of daily living (ADL) (such as washing, dressing [3]) and instrumental ADL (IADL) (such as preparing meals [3]) by their own [4, 5]. In the Netherlands, however, older adults who need support in functional activities can receive community care, which comprises nursing care (e.g., personal care) and domestic services (e.g., cleaning). Nursing care is provided by teams with a mix of community care professionals (e.g., bachelor- and vocationally-educated nurses, certified nurse assistants, and helping aids), while domestic support workers are more involved in providing domestic services [6]. Bachelor-educated community nurses fulfil a lead role in the teams since they coordinate the care process and conduct needs assessments to determine which and how much care is necessary.

It is important that community care professionals encourage older adults to engage in functional activities [7] and to be active during daily care activities. For example, by using verbal instructions or assistive devices during bathing, older adults can wash themselves, instead of professionals doing it for them [8]. However, community care professionals do not always have the competencies, that is, attitude, knowledge and skills to encourage older adults in functional activities [9, 10]. They are often performing care activities on behalf of older adults instead of encouraging them to perform these activities as independently as possible [10, 11]. Further insights are needed into the current behavior of professionals and into the factors, the professionals encounter as barriers.

Existing questionnaires generally focus only on measuring the role of nurses in, for example, encouraging physical activity [11, 12]. However, for measuring the role of nurses in encouraging functional activities, the MAastrIcht Nurses Activities INventory (MAINtAIN) questionnaire was developed. This questionnaire measures the behavior and barriers of nurses in nursing homes in encouraging functional activities [12]. The questionnaire consists of two scales, namely the Behaviors scale for measuring the perceived behavior of professionals in encouraging functional activities, and the Barriers scale for measuring the related barriers to this behavior. The Behaviors scale comprises three subscales with 19 nine-point scaled items measuring the extent to which professionals stimulate residents in performing ADL (e.g., dressing), IADL (e.g., making the bed), and miscellaneous activities (e.g., encouraging physical activity as part of the care plan). The three subscales showed good internal consistency, Cronbach’s alpha ranged from .77 to .83 [13]. The MAINtAIN-Barriers scale comprises 33 nine-point scaled items measuring barriers and facilitators related to the clients’ context, as well as the professional, social (i.e., the team functioning), and organizational (i.e., how things work within the organization) contexts [14]. The MAINtAIN was developed based on Restorative Care literature, an approach to improve functions of older adults [15–19] and on literature about evidence-based nursing interventions and innovations [20, 21]. The usability and content validity of the MAINtAIN were established involving experts, nursing staff, and residents [12].

The MAINtAIN seemed promising for use in nursing home care. The usability study indicated that completing the questionnaire was not difficult and that it had clear items and response options. The number of missing values was low and a floor or ceiling effect was shown for a few items [12]. However, this setting is different from the community care setting. For example, while the nursing home professionals provide the care in teams at the nursing home wards, the community care professionals provide care at the clients’ homes [22]. Therefore, adaptation of the MAINtAIN is necessary to make it applicable and valid for using it for community care professionals [23]. In the current study, the MAINtAIN questionnaire is adopted. The objective is to create a modified version that is suitable for measuring behavior and barriers of community care professionals in encouraging functional activities of clients in the community care setting. The overall aims are to assess the content validity, construct validity, and internal consistency of the modified version. The specific aims are to answer the research questions:

1. Which items of the MAINtAIN questionnaire should be adapted to make it appropriate and feasible for the community care setting, and what is the content validity of the adapted questionnaire?
2. What is the construct validity and internal consistency of the adapted questionnaire?

## Phase 1. Assessing the appropriateness and feasibility of the MAINtAIN and assessing the content validity of the adapted questionnaire

### Methods

A prospective study design using qualitative and quantitative methods to collect data was conducted. Data were collected in two phases. During phase one, the MAINtAIN was assessed on appropriateness and feasibility by community nurses, and the adapted questionnaire was assessed on content validity by research experts and community care professionals. Data were collected between February 2017 and July 2017.

#### Measures

##### MAINtAIN-questionnaire

The questionnaire consists of two scales, namely the Behaviors scale for measuring the perceived behavior of professionals in encouraging functional activities, and the Barriers scale for measuring the related barriers to this behavior. The Behaviors scale comprises three subscales with 19 nine-point scaled items measuring the extent to which professionals stimulate residents in performing ADL (e.g., dressing), IADL (e.g., making the bed), and miscellaneous activities (e.g., encouraging physical activity as part of the care plan). The three subscales showed good internal consistency, Cronbach’s alpha ranged from .77 to .83 [13]. The MAINtAIN-Barriers scale comprises 33 nine-point scaled items measuring barriers and facilitators related to the clients’ context, as well as the professional, social (i.e., the team functioning), and organizational (i.e., how things work within the organization) contexts [14].

##### Groningen activity restriction scale (GARS)

The GARS measures disability in ADL and IADL. The self-report questionnaire comprises two subscales, measuring ADL (11 items) and IADL (seven items), with four response options per item, ranging from one = able to perform the activity without any difficulty, to four = unable to perform the activity independently. The total score for disability ranges from 18 to 72, with higher scores indicating more disability, and the Cronbach’s alpha for the subscales has shown to be .82 and .80, respectively [24, 25].

##### COnsensus-based standards for the selection of health measurement instruments (COSMIN)-checklist

The checklist [26] comprises 12 boxes to assess if studies on measurement properties meet the requirements and to assess the different measurement properties included, such as the internal consistency, reliability and construct validity. An additional box evaluates the quality of a study on interpretability. Several experts in the field of measurement with different backgrounds were involved in the development of the COSMIN checklist.

#### Participants and procedure

Seven bachelor-educated community nurses were invited to assess the appropriateness and feasibility of the MAINtAIN. It was expected that their lead role in the community care teams made them most suitable for this assessment. Convenience sampling was used to recruit the nurses, in collaboration with two managers of two long-term care organizations that provide community care in the South of the Netherlands. Inclusion criteria were: 1) employed as a bachelor-educated nurse and 2) not involved in the data collection of another study. The managers of the organizations selected the community nurses, who were each in charge of one community care team. All the invited nurses *(N* = 7) participated in the study. Six nurses were female, five were bachelor-educated and two had obtained a master’s degree. The median age was 34 years old (range 26–56), they had a median work experience of 14 years (range 7–38), and the median work hours per week were 32 h (range 24–36). Next, research experts and community care professionals were invited to assess the content validity of the adapted questionnaire. Nine research experts from Maastricht University and Zuyd University of Applied Sciences were invited to participate in this phase, including Authors SFM, EVR, PMGE and SMGZ, and all the nine research experts participated in the study. Three research experts were male and six were female, three research experts also had a background in nursing. Furthermore, convenience sampling was used to recruit community care professionals from another long-term care organization that provided community care in the Netherlands. Inclusion criteria were: 1) employed as a bachelor- or vocationally-educated nurse, certified nurse assistant, helping aid or nursing student; and 2) not involved in the data collection of another study. Based on these criteria, the managers of the organizations selected 20 community care professionals. Eighteen of the 20 community care professionals participated in the study. The community care professionals were all female: five professionals worked as a bachelor-educated nurse, two professionals worked as a vocationally-educated nurse, and eleven professionals worked as a certified nurse assistant or helping aid. The median age was 46 years (range 25–60), the median work experience was 18 years (range 4–41), and the median work hours per week was 27 (range 20–36).

To assess the appropriateness (i.e., the perceived fit or relevance for a given setting) and feasibility (i.e., the extent to which it can be successfully used or carried out within a given setting) [27] of the MAINtAIN, the community nurses were invited to attend four meetings. Each meeting followed the same procedure, and different items were assessed. First, the nurses could individually assess each item on the appropriateness and feasibility for community care. They could propose adaptations for the formulation and wording, suggest which specific nursing home items were not relevant, and suggest additional relevant items to measure the behavior and barriers in the community care setting. Second, a group discussion [28] took place during the meeting, regarding the appropriateness and feasibility of the items. One researcher (author RGMV) was the moderator and took additional field notes [28]. All the comments of the individual nurses were gathered after the meetings.

To assess the content validity of the adapted MAINtAIN, the nine research experts received the questionnaire via e-mail. To assess the content validity, they used the COSMIN checklist [26]. They assessed the content validity by reviewing the name, description, the instructions, the response options, and the relevance (i.e., whether all the included items were relevant), comprehensiveness (i.e., whether all key items were included), and comprehensibility (i.e., whether the items were understood and appropriately worded). The 20 community care professionals received a paper-based version of the adapted MAINtAIN. They were invited to assess the content validity by completing the questionnaire and reviewing the comprehensibility. Half of the professionals received items formulated based on the plural pronoun, “We” (as in the original MAINtAIN questionnaire; in other words, “In my team, we closely follow”), and half of them with items formulated based on the singular pronoun “I.” This was done to assess if the adapted formulation would better fit the context, since community care professionals individually perform care activities at the clients’ homes. Next to this, background characteristics of the nurses, the community care professionals, and the research experts (e.g., age and years of experience) were assessed.

#### Data analysis

The comments of the community nurses, the research experts, and the community care professionals, as well as the field notes from the group discussions with the nurses were gathered and summarized. Two researchers (Authors RGMV and GJJB) evaluated the comments and discussed the appropriateness and feasibility until consensus was reached and, if needed, adaptations were incorporated [29]. The additional items for the Behaviors scale (i.e., items that were not in the MAINtAIN but suggested by the nurses to be important for the adapted questionnaire for community care) were clustered according to the Groningen Activity Restriction Scale (GARS) [3]. The additional relevant items for the community care were clustered in either ADL or IADL, based on the clustering of the GARS. The additional items for the Barriers scale were clustered according to the original domains of the MAINtAIN questionnaire.

### Results

#### Adaptation of the MAINtAIN and content validity of the MAINtAIN-C

The MAINtAIN was adapted in formulation (changed to the singular pronoun, “I,” to better fit the context), in verbs and wording to make it suitable for community care. Furthermore, specific nursing home items were excluded (i.e., two items for the Behaviors scale and four items for the Barriers scale), and relevant items for community care were added (i.e., four items for the Behaviors scale and four items for the Barriers scale). Furthermore, the name changed to the MAastrIcht Nurses Activities INventory for Community Care (MAINtAIN-C). See Table 1 for an overview of all the adaptations. The original MAINtAIN included both facilitators and barriers but was used to measure barriers [14] which is why we reversed the positively formulated items and interpreted all items as barriers.

**Table 1:** Adaptations to the MAINtAIN questionnaire

Aspect	Adaptations
Name	The name of the questionnaire changed to the MAastrIcht Nurses Activities INventory for Community Care (MAINtAIN-C).
Formulation	The formulation of all the items changed to the singular pronoun, “I,” to better fit the context, since community care professionals individually perform care activities. This was further supported by comments of two community care professionals, stating that they found it difficult to answer the questions on behalf of their team members.
Verbs	The IADL items changed to the verbs, “I discuss” (MAINtAIN-C, instead of “I encourage” (original MAINtAIN)), since these activities are performed by domestic support workers, who are not the end users of the MAINtAIN-C.
Wording	The wording of items was adapted to make them suitable for community care; for example, from “We prepare sandwiches for residents, even if they can do this themselves” (original MAINtAIN Behaviors, item 9), to “I discuss with clients if they can prepare their meals independently” (adapted MAINtAIN Behaviors item 12).
Excluded items	For the Behaviors scale, two specific nursing home items were excluded; for example, item 10: “We encourage residents to help set and clear the table.” For the Barriers scale, four specific nursing home items were excluded from the questionnaire since they were considered less relevant for community care; for instance, item 8: “Residents on my ward consider it perfectly normal to have others move them instead of moving about themselves.”
Added Items	For the Behaviors scale, four specific community care items were added; for instance, new item 14: “I advise clients about the added value of consulting other disciplines (e.g., physical therapy) to encourage the independent performance of ADLs, since they were considered relevant for measuring behavior in the community care setting.” For the Barriers scale, four specific community care items were added; for example, new item 10: “An overburdened family or informal caregiver limits clients in performing ADLs and IADLs independently.”
Order	The order of the items was changed to cluster activities as much as possible, based on the clustering of the GARS for the Behaviors scale [3], as well as the original clustering of the original MAINtAIN for the Barriers scale.
Number of items	For the Behaviors scale, the number of items changed from 19 to 20. For the Barriers scale, the number of items remained the same as the original MAINtAIN (33 items).

#### Final version of the MAINtAIN-C

See Table 2 and Table 3 for an overview of the final items of the MAINtAIN-C. The MAINtAIN-C Behaviors scale contained 20, 9-point scaled items and assessed the degree to which community care professionals perceived to encourage functional activities related to ADL (11 items), IADL (5 items), and general activities (4 items). Answer options ranged from “one = never, to five = sometimes, to nine = always.” The MAINtAIN-C Barriers scale assessed related barriers in encouraging functional activities of community-dwelling older adults, containing 33, 9-point scaled items, with factors relating to the clients’ context (10 items), as well as the professionals (10 items), social (i.e., the team functioning) (6 items), and organizational (7 items) contexts. Answer options ranged from “one = never, to five = sometimes, to nine = always” and “one = completely disagree, five = neither agree nor disagree, nine = completely agree.”

**Table 2:** MAINtAIN-C Behaviors

Items	
*ADL-activities*	
1. I closely follow the extent to which clients are able to perform their own ADLs.	
2. I encourage clients to perform their own ADLs as much as possible.	
3. I closely follow the extent to which assistive devices are available and adequately used by clients.	
4. I advise clients about the added value of consulting other disciplines (for example physical therapy) in order to encourage the independent performance of ADLs.	
5. I discuss with new clients if a meal can be eaten independently.	
6. I encourage clients to independently dress and undress.	
7. I complement clients when they dress and undress themselves.	
8. I closely follow the extent to which clients are to move about within their home.	
9. I encourage clients to participate in activities outside their home.	
10. I encourage clients to independently wash and dry themselves.	
11. I encourage clients to use the toilet independently.	
*IADL-activities*	
12. I discuss with clients if they can prepare their meals independently.	
13. I discuss with clients if they can do their shopping independently.	
14. I discuss with clients if they can wash their clothes independently.	
15. I discuss with clients if they can do ‘light housework’ independently, for example washing/drying dishes.	
16. I discuss with clients if they can mop the bathroom independently after getting washed.	
*General activities*	
17. I discuss with clients which activities they used to do and they still can perform (ADLs, IADLs, and social activities).	
18. I discuss with clients which activities (ADLs, IADLs, and social activities) they would really like to perform themselves.	
19. I encourage the clients’ family and informal caregivers to promote self-reliance in clients.	
20. If ‘encourage self-reliance and independence’ is included in the care plan, then I follow this.	

*The MAINtAIN-C was translated from Dutch to English in two steps. First, one bilingual independent translator working as a researcher and professional translator, who also translated the original MAINtAIN questionnaire, and one author (RGMV), both translated the MAINtAIN-C into English. Second, the differences between these two versions and the original MAINtAIN were discussed by two researchers (RGMV, GJJB) until consensus was reached on a final version* [29]*.*

**Table 3:** MAINtAIN-C Barriers

*Context of the clients*	
1. Clients are often able to control factors that influence their situation.	
2. Clients are often able to perform ADLs more independently than they now do.	
3. I see that encouraging physical activity has a positive effect on clients.	
4. The capability of family or informal caregivers to encourage clients in the independent performance of ADLs and IADLs is sufficient.	
5. Clients are afraid to walk on their own, without help from others.	
6. Clients ask for help with ADLs so that they can get extra attention.	
7. Family or informal caregivers expect the nurses and nurse assistants to take over the activities that clients themselves can still perform.	
8. Clients do not want to perform activities themselves such as bathing or opening/closing curtains even if they still can.	
9. Financial limitations restrict clients in performing ADLs and IADLs independently.	
10. An overburdened family or informal caregiver limits clients in performing ADLs and IADLs independently.	
*Context of the professionals*	
11. I think that organizing my work so that clients are ready on time is more important than clients performing ADLs independently.	
12. I am afraid that clients will hurt themselves if I encourage them to walk alone.	
13. It is primarily the responsibility of the physical therapist or occupational therapist to encourage clients to perform activities.	
14. Within my team, we think that it is important to encourage clients to perform ADLs as much as possible independently.	
15. Within my team, we think that it is our task to inform the family or informal caregivers about the importance of clients performing activities independently.	
16. I expect that encouraging ADLs and IADLs has no effect on how clients function.	
17. Within my team, sufficient expertise is available to encourage clients to be as independent as possible in performing ADLs (such as bathing, moving about).	
18. Encouraging independence as much as possible in clients’ ADLs, IADLs and social activities gives me less time for other things.	
19. I find it difficult to encourage clients to be self-reliant and independent.	
20. If I want, I am able to allow clients to perform ADLs and IADLs more independently.	
*The social context (the team functioning)*	
21. Within my team, the collaboration with experts (for example occupational or physical therapists) is not good enough to encourage clients in performing ADLs as independently as possible.	
22. I can count on enough support from my colleagues when I allow clients to perform ADLs and IADLs as independently as possible.	
23. The manager of my team considers it important that clients perform ADLs and IADLs as independently as possible.	
24. I speak to my colleagues when I hear that they perform activities that clients can still perform themselves.	
25. The team discusses how we can encourage clients to perform ADLs and IADLs as independently as possible.	
26. Within my team, it is our routine to take over the ADLs and IADLs (such as making sandwiches) for our clients.	
*The organizational context*	
27. My organization is not geared towards involving clients in the performance of ADLs and IADLs (such as independently bathing and dressing or preparing a meal).	
28. In my organization, there are enough people available with knowledge about how to encourage self-reliance and independent performance of activities by clients.	
29. My organization offers the possibility to follow internal or external courses that focus on encouraging clients’ physical activity.	
30. In my organization, we do not have agreements or guidelines concerning how we can encourage clients’ physical activity.	
31. I have inadequate time to activate clients to be self-reliant because of the needs assessment determined by the community nurse in my team.	
32. Encouraging self-reliance and independence has a high priority in my organization.	
33. There is a structural shortage of staff available to encourage clients to perform ADLs and IADLs (such as independently bathing and dressing or preparing a meal) as independently as possible.	

## Phase 2. Assessing the construct validity and internal consistency of the final adapted questionnaire

### Methods

During phase two, the psychometric properties of the final adapted questionnaire were assessed in a sample of community care professionals. Data were collected between September 2017 and March 2018.

#### Participants

The team members of the seven community care nurses that participated in phase one were recruited to assess the construct validity and internal consistency. There were no additional inclusion criteria for the team members. In total, 80 community care professionals were eligible for the study.

#### Data collection

Before completing the questionnaire, the community care professionals received a link with a personal account to log in via an online program in which they could complete the questionnaire and they were allowed to provide comments to the questions. Furthermore, background characteristics (e.g., age and years of experience) were assessed. Between two and 4 weeks after the initial invitation, reminder emails to complete the questionnaire were sent to the non-respondents.

#### Data analysis

Data were analyzed using IBM SPSS Statistics 25.0 for Windows [30]. For the Barriers scale, the scores of the positively formulated items were reversed so that higher scores always indicate stronger experienced barriers. Items were checked for missing values. For each respondent, the missing values were imputed with the average score of all respondents on all items in that scale, if at least 80% of the items of that scale had been completed by the respondent [31]. Descriptive statistics were performed to give an overview of the study sample and to check for outliers and floor and ceiling effects. An Exploratory Factor Analysis (EFA) assessed the construct validity. Principal axis factoring (PAF) with direct Oblimin (oblique) rotation was used to explore the structure of the scales. PAF was used since we attempted to identify latent constructs (factors) that could explain the pattern of item-item correlations, rather than decomposing the data into a set of linear variates to explain as much variance as possible, as in principal component analysis (PCA). The direct Oblimin technique was used to allow the factors to be correlated with each other. For the Kaiser-Meyer-Olkin measure of sampling adequacy we accepted a criterion of above 0.50 [32] and for the Bartlett’s test of sphericity, we required significance at the 5% level, meaning rejection of the null hypothesis that all item-item correlations are zero [33]. The internal consistency was assessed in terms of Cronbach’s Alpha [34] and an item analysis was further performed by evaluating the corrected item-total correlations, based on a tentative criterion of 0.30, as an acceptable correlation [35].

### Results

#### Sample characteristics

All the invited community care professionals (*N* = 80) returned the questionnaire. Missing data of nine respondents for the MAINtAIN-C Behaviors were imputed (of whom six respondents had one missing item, and three respondents had two missing items). Missing data of 15 respondents for the MAINtAIN-C Barriers were imputed (of whom 9 respondents had 1 missing item, and 6 respondents had 2 missing items). One respondent had more than 20% missing on the Behaviors scale and was excluded from the analyses on the complete MAINtAIN-C scale. See Table 4 for an overview of the sample characteristics of the community care professionals (*N* = 79).

**Table 4:** Sample characteristics of the community care professionals *(N = 79)*

		*N*	%
Gender	Female	77	(97)
Profession	Bachelor educated nurse	7	(9)
	Vocationally educated nurse	16	(20)
	Certified Nurse Assistant / Helping Aid / Nursing Student	56	(71)
Education	Bachelor of Nursing	8	(10)
	Vocational training	23	(29)
	Secondary training	48	(61)
		Median	Range [min-max]
Age (years)		47.8	45.2 [20–65] ^a^
Work experience (years)		18.0	40.6 [1–42] ^b^
Working hours per week		24.0	28.0 [8–36]

^a^*Based on N = 78, due to missing data.* ^b^*Based on N = 77, due to missing data*

### Construct validity and internal consistency

#### Factor analysis for the MAINtAIN-C behaviors

The EFA was carried out on the final MAINtAIN-C Behaviors questionnaire, which contained 20 items. This yielded a potential four-factor solution (eigenvalue > 1 and scree plot; see Supplementary Table 1, Additional File 1). Before rotation, the first factor accounted for 44% variance, the second for 13%, the third for 7%, and the fourth for 5%, while all further factors each explained less than 5%. After Oblimin rotation, no meaningful pattern in the loadings could be determined. Then, a three-factor solution was performed, and after Oblimin rotation, all the eight items containing the verbs, “I discuss” (items 5, 12–18), loaded strongly on factor 2 (F2) and much less on factor 1 (F1) and factor 3 (F3). The other 12 items loaded strongest either on F1 or on F3. No clear and interpretable pattern in the loadings on F1 and F3 could be determined.

Furthermore, F1 and F3 correlated − 0.46 with each other (see Supplementary Table 1, Additional File 1). This suggested a two-factor solution. After Oblimin rotation, all items containing the verb, “I discuss,” except item 18, loaded strongly on F2, all the other items loaded strongly on F1. Item 18 had nearly the same loading on F1 and F2. The factor-to-factor correlation was − 0.495 (implying a positive correlation between the two item subsets, see the signs of the factor loadings in Supplementary Table 1, Additional File 1. Therefore, we performed a reliability analysis on F1 (12 items) and F2 (8 items including item 18).

The Cronbach’s alpha for internal consistency of F1 was .88, with item-total correlations for all items above 0.35. The Cronbach’s alpha for F2 was .92, with item-total correlations for all items above 0.57. The Pearson correlation between the mean scores on F1 and F2 was 0.61, indicating a strong, positive relationship. Therefore, a single-factor model was also performed (see Table 5).

**Table 5:** Factor loadings after Oblimin rotation in the EFA* of the MAINtAIN-C scale (*N* = 79)

MAINtAIN-C Behaviors		MAINtAIN-C Barriers		
One-factor solution		Two-factor solution		
Items	Factor 1	Items	Factor 1	Factor 2
		*Context of the clients*		
1	.567	1	**.346**	−.188
2	.652	2	−.060	**.556**
3	.617	3	**.252**	−.222
4	.671	4	**.329**	−.103
5	.651	5	−.092	**.480**
6	.647	6	.139	**.699**
7	.306	7	.130	**.542**
8	.737	8	.026	**.656**
9	.575	9	.151	**.571**
10	.440	10	.075	**.569**
11	.654	*Professional context*		
12	.683	11	**.243**	−.100
13	.682	12	**.402**	.080
14	.800	13	**.231**	−.036
15	.838	14	**.385**	−.065
16	.639	15	**.566**	.000
17	.690	16	**.116**	.109
18	.666	17	**.615**	.191
19	.686	18	**.421**	−.047
20	.384	19	**.288**	.030
		20	.042	**−.146**
		*Social context: the team functioning*		
		21	**.586**	.052
		22	**.696**	.216
		23	**.460**	.055
		24	**.235**	−.180
		25	**.540**	.010
		26	**.509**	.207
		*Organizational context*		
		27	**.594**	.068
		28	**.489**	.076
		29	**.378**	.040
		30	**.523**	−.208
		31	**.202**	.036
		32	**.586**	.053
		33	**.455**	−.024
		Factor Correlations		
		Factors	1	2
		1	1	−.001
		2	−.001	1

*The EFA was conducted using principal axis factoring and a direct Oblimin (oblique) rotation; factor loadings in boldface are the highest loading of that item

The Cronbach’s alpha of the total scale was .92, with item-total correlations ranging from 0.27 to 0.81. The Kaiser-Meyer-Olkin measure of sampling adequacy was .87 and Bartlett’s test of sphericity was significant (*p* < .05). We opt for a single-factor model, because of the strong positive correlation in the two-factor model, the good internal consistency of the single-factor model, and the theoretical fit of all the items in one scale. The sum score on this total scale for each respondent varying from 20 to 180, indicates the degree to which the respondent is perceived to encourage functional activities. See Additional File 2 for the complete MAINtAIN-C questionnaire.

#### Factor analysis for the MAINtAIN-C barriers

The EFA carried out on the MAINtAIN-C Barriers questionnaire with 33 items, led to a 10-factor solution according to the eigenvalue > 1 criterion, but the scree-plot suggested four or possibly three factors. Before rotation, the first factor accounted for 18% variance, the second for 10%, the third for 7%, and the fourth for 5%, while all further factors each explained less than 5%. Therefore, both a four-factor solution and a three-factor solution were obtained. In both cases, after Oblimin rotation, no meaningful pattern in the loadings could be determined (see Supplementary Table 2, Additional File 1).

Therefore, a two-factor solution was obtained and, after Oblimin rotation, 25 items loaded on F1, of which 22 items related to the professional, social, and organizational contexts. Eight items loaded on F2, of which 7 items were describing barriers related to the clients’ context (see Table 5). Misfitting items were items 1, 3, and 4 (loaded stronger on F1, but are about the clients’ context), item 20 (loaded stronger on F2, but is about the professional context) and item 16 (loaded less than 0.20 on F1 and almost equally high on both factors). The correlation between F1 and F2 was − 0.001.

We performed a reliability analysis on all items loading the highest on F1, except the misfitting items 1, 3, 4, and 16 (see Table 5). This resulted in a scale of 21 items, with a Cronbach’s alpha of .83 and item-total correlations ranging from 0.22 to 0.59. We also performed a reliability analysis on all items loading highest on F2, except the misfitting item 20 (i.e., 7 items in total; see Table 5), which gave a Cronbach’s alpha of .78 and item-total correlations ranging from 0.39 to 0.62. The correlation between the mean scores on the two subscales (i.e., F1 and F2 without the misfitting items) was 0.10, indicating a very weak to absent (linear) relationship.

To compare, we also computed correlations between the mean scores on the four predefined domains (i.e., factors related to the clients’ context, as well as the professional, social, and organizational contexts). The correlations between the different contexts ranged from 0.16 (between the clients’ context and the organizational context) to 0.61 (social and the organizational context). These correlations further supported the reduction to two subscales, one for the clients’ context and one for the other three contexts.

We, therefore, opt for a two-factor solution with 7 items related to the clients’ context, with good internal consistency (Cronbach’s alpha: .78), and 21 items related to the professional, social, and organizational contexts, with good internal consistency (Cronbach’s alpha: .83). The Kaiser-Meyer-Olkin measure of sampling adequacy for the two-factor solution was .58 and Bartlett’s test of sphericity was significant (*p* < .05). The sum score per subscale—varying from 7 to 63 for the clients’ context and from 21 to 189 for the professional, social, and organizational contexts, for each respondent—indicates the degree to which the respondent is perceived to experience barriers in stimulating functional activities. See Additional File 2 for the complete MAINtAIN-C questionnaire.

## Discussion

In the first phase of this study, the MAINtAIN questionnaire for the nursing home setting was adapted for the community care setting. This resulted in the MAINtAIN-C questionnaire, consisting of two scales to measure perceived behavior (20 items) and barriers (33 items) of community care professionals, in encouraging functional activities of clients in the community care setting. During the second phase, the construct validity and internal consistency of the MAINtAIN-C were assessed. This resulted in the Behaviors scale (20 items), which measures the perceived behavior of community care professionals in encouraging functional activities, showing good internal consistency (Cronbach’s alpha: .92). The Barriers scale measures barriers in encouraging functional activities related to two dimensions: 1) the clients’ context (7 items), with good internal consistency (Cronbach’s alpha: .78); and 2) the professional, social, and organizational contexts (21 items), showing good internal consistency (Cronbach’s alpha: .83).

Although no factor analysis had been performed in the original study in which the MAINtAIN was presented, we had expected that the initial theoretical clustering of the original MAINtAIN Behaviors (i.e., ADL, IADL, and general activities) would also be present in the adapted MAINtAIN-C questionnaire [12, 13]. However, in the adapted MAINtAIN-C Behaviors scale, all items measured largely the same construct. It could be that the distinction between activities is more clear in nursing homes than in community care, since the care in nursing homes is primarily focuses on providing assistance in ADL [36]. It is likely that community care professionals interpret the encouragement of functional activities as all activities (i.e., ADL, IADL, and general activities) that directly take place at the clients’ home. Only a distinction between the items with the verbs “I discuss,” and the other items emerged, but the correlation between these two different factors was strong. One could argue that community care professionals might view the “I discuss” items differently, since these relate more to their direct personal behavior, than the other items.

For the adapted MAINtAIN-C Barriers scale, it was expected that the differences between the four domains (i.e., barriers related to the clients’ context, as well as the professional, social, and organizational contexts) would emerge as in the initial theoretical clustering of the MAINtAIN [14]. Instead, two dimensions emerged, namely the clients’ context versus the other three contexts. This contradicts other studies reporting that barriers often relate to various domains [37, 38]. On the other hand, studies on promoting physical activity or function also report on barriers as a combination of professional and organizational factors, such as lack of (quality of) time [19, 39–41] lack of training and education [40, 41] and lack of protocols [39, 41] versus patient-related factors, such as lack of motivation of the patient [19, 40].

### Limitations

This study has some methodological limitations. First, a modest sample size was used for conducting the EFA. Therefore, the statistical findings presented should be interpreted with caution and replicated in a larger sample. Next, only bachelor-educated community nurses were involved in the adaptation process during the first phase of the study, while community care professionals are the end users. However, the leading role of community nurses within community care might have warranted the inclusion of important behaviors and barriers. We also used a convenience sample of community care professionals to assess the content validity. Whether the results found in this study are generalizable to the community care setting at large remains to be demonstrated.

The content validity of the MAINtAIN-C was assessed via a paper-based version of the questionnaire, while the construct validity and internal consistency were assessed via an online version of the MAINtAIN-C. This might have influenced the usability, but we tried to minimize this by providing clear instructions in the online program. Furthermore, the MAINtAIN-C questionnaire relies on self-reported data, which means that the reported perceived behavior may not necessarily be the same as their actual behavior in practice. Prior studies indicated, for example, that professionals in nursing homes perceive to encourage ADL often [13] while observations indicate that the majority of residents are largely inactive during the day [42]. Although we tried to minimize bias by informing the respondents about the anonymous administration of the questionnaire, social desirability might have influenced the response.

### Implications

The MAINtAIN-C is, to the best of our knowledge, the first questionnaire for assessing behavior and barriers of community care professionals in encouraging functional activities. The MAINtAIN-C can be used to provide insight into the behavior and barriers of community care professionals. Since these professionals are often used to perform functional activities on behalf of older adults, the MAINtAIN-C can be a useful learning instrument to display the possible difference between perceived and actual behavior in practice. Furthermore, insights on the perceived behavior and barriers of community care professionals in encouraging functional activities can be useful for researchers, managers, community nurses, and other community care professionals. Strategies to promote certain behavior and tackle the barriers can be implemented, to foster a change in behavior. Adopting these strategies within daily procedures and policies within community care, could eventually lead to increased or maintained functional activity among older adults living in the community.

## Conclusions

The MAINtAIN-C seems promising to assess the behavior and barriers of community care professionals in encouraging functional activities. The results of this study also provide guidance for further research in a larger sample, to obtain more insight into the psychometric properties such as the ability of the MAINtAIN-C to measure changes in encouraging functional activity over time (responsiveness) and the degree of consistency of the MAINtAIN-C data obtained by the same rater (intra-rater reliability).

## Supplementary information

**Additional File: 1 Supplementary Table 1 and Supplementary Table 2.** show the complete factor loadings on the MAINtAIN-C Behaviors and Barriers scales.

**Additional File 2.** The MAINtAIN-C questionnaire. The MAINtAIN-C shows the MAINtAIN-C Behaviors scale and MAINtAIN-C Barriers scale

## Data Availability

The datasets used and/or analyzed during the current study are available from the corresponding author on reasonable request.

## Abbreviations

ADL: Activities of daily living
EFA: Exploratory factor analysis
COSMIN: Consensus-based standards for the selection of health status measurement instruments
GARS: Groningen activity restriction scale
IADL: Instrumental activities of daily living
MAINtAIN: Maastricht nurses activities inventory
MAINtAIN-C: Maastricht nurses activities inventory for community care

## References

[1] World Health Organization. World report on ageing and health. Geneva: World Health Organization; 2015.

[2] Plaisier I, Verbeek-Oudijk D, de Klerk M (2016). Developments in home-care use. Policy and changing community-based care use by independent community-dwelling adults in the Netherlands. Health Policy.

[3] Kempen GIJM, Doeglas DM, Suurmeijer TPMB: Groningen activity restriction scale (GARS): een handleiding. UMCG / Rijksuniversiteit Groningen, Research Institute SHARE 2012, Tweede herziene druk**.**.

[4] Albrecht GL, Snyder SL, Bickenbach J, Mitchell DT, Schalick WO (2006). Encyclopedia of disability.

[5] Connolly D, Garvey J, McKee G (2017). Factors associated with ADL/IADL disability in community dwelling older adults in the Irish longitudinal study on ageing (TILDA). Disabil Rehabil.

[6] Genet N, Boerma W, Kroneman M, Hutchinson A, Saltman R: Home care across Europe: case studies. 2013.

[7] Winkel A, Langberg H, Wæhrens EE (2015). Reablement in a community setting. Disabil Rehabil.

[8] Resnick B, Galik E, Boltz M (2013). Function focused care approaches: literature review of progress and future possibilities. J Am Med Dir Assoc.

[9] Legg L, Gladman J, Drummond A, Davidson A (2016). A systematic review of the evidence on home care reablement services. Clin Rehabil.

[10] Aspinal F, Glasby J, Rostgaard T, Tuntland H, Westendorp RG (2016). New horizons: Reablement-supporting older people towards independence. Age Ageing.

[11] Whitehead PJ, Worthington EJ, Parry RH, Walker MF, Drummond AE (2015). Interventions to reduce dependency in personal activities of daily living in community dwelling adults who use homecare services: a systematic review. Clin Rehabil.

[12] Kuk N, Zijlstra GAR, Bours GJJW, Hamers JPH, Kempen GIJM (2016). Development and usability of the MAINtAIN, an inventory assessing nursing staff behavior to optimize and maintain functional activity among nursing home residents: a mixed-methods approach. BMC Health Serv Res.

[13] Kuk N, den Ouden M, Zijlstra GAR, Hamers JPH, Kempen GIJM, Bours GJJW (2017). Do nursing staff encourage functional activity among nursing home residents? A cross-sectional study of nursing staff perceived behaviors and associated factors. BMC Geriatr.

[14] Kuk NO, Zijlstra GR, Bours GJ, Hamers JP, Tan FE, Kempen GI (2018). Promoting functional activity among nursing home residents: a cross-sectional study on barriers experienced by nursing staff. J Aging Health.

[15] Resnick B, Rogers V, Galik E, Gruber-Baldini AL (2007). Measuring restorative care provided by nursing assistants: reliability and validity of the restorative care behavior checklist. Nurs Res.

[16] Resnick B, Gruber-Baldini AL, Zimmerman S, Galik E, Pretzer-Aboff I, Russ K, Hebel JR (2009). Nursing home resident outcomes from the res-care intervention. J Am Geriatr Soc.

[17] Galik EM, Resnick B, Pretzer-Aboff I (2009). ‘Knowing what makes them tick’: motivating cognitively impaired older adults to participate in restorative care. Int J Nurs Pract.

[18] Resnick B, Simpson M, Galik E, Bercovitz A, Gruber-Baldini AL, Zimmerman S, Magaziner J (2006). Making a difference: nursing assistants' perspectives of restorative care nursing. Rehabil Nurs.

[19] Resnick B, Petzer-Aboff I, Galik E, Russ K, Cayo J, Simpson M, Zimmerman S (2008). Barriers and benefits to implementing a restorative care intervention in nursing homes. J Am Med Dir Assoc.

[20] Bulechek GM, Butcher HK, Dochterman JM, Wagner CM. Nursing Interventions Classification (NIC) (6th ed.). St. Louis: Elsevier Mosby; 2013.

[21] Fleuren MAH, Paulussen TGWM, Van Dommelen P, Van Buuren S (2014). Towards a measurement instrument for determinants of innovations. Int J Qual Health Care.

[22] Wysocki A, Butler M, Kane RL, Kane RA, Shippee T, Sainfort F (2015). Long-term services and supports for older adults: a review of home and community-based services versus institutional care. J Aging Soc Policy.

[23] Beaton DE, Bombardier C, Guillemin F, Ferraz MB (2000). Guidelines for the process of cross-cultural adaptation of self-report measures. Spine.

[24] Kempen GIJM, Miedema I, Ormel J, Molenaar W (1996). The assessment of disability with the Groningen activity restriction scale. Conceptual framework and psychometric properties. Soc Sci Med.

[25] Kempen GIJM, Steverink N, Ormel J, Deeg DJH (1996). The assessment of ADL among frail elderly in an interview survey: self-report versus performance-based tests and determinants of discrepancies. J Gerontol Ser B Psychol Sci Soc Sci.

[26] Mokkink LB, Terwee CB, Patrick DL, Alonso J, Stratford PW, Knol DL, Bouter LM, de Vet HCW (2010). The COSMIN checklist for assessing the methodological quality of studies on measurement properties of health status measurement instruments: an international Delphi study. Qual Life Res.

[27] Proctor E, Silmere H, Raghavan R, Hovmand P, Aarons G, Bunger A, Griffey R, Hensley M (2011). Outcomes for implementation research: conceptual distinctions, measurement challenges, and research agenda. Adm Policy Ment Health Ment Health Serv Res.

[28] Flick U (2009). An introduction to qualitative research.

[29] Polit DF, Beck CT (2012). Nursing research : generating and assessing evidence for nursing practice.

[30] IBM (2017). IBM SPSS Statistics for Windows, Version Q3 25.0.

[31] Béland S, Pichette F, Jolani S (2016). Impact on Cronbach’s α of simple treatment methods for missing data. Quant Methods Psychol.

[32] Kaiser HF (1974). An index of factorial simplicity. Psychometrika.

[33] Field A. Discovering statistics using IBM SPSS Statistics. 4th ed. London: SAGE Publication Lda; 2013.

[34] Cronbach LJ (1951). Coefficient alpha and the internal structure of tests. Psychometrika.

[35] Nunnally JC, Bernstein IH. Psychometric theory. 3rd ed. New York: McGraw-Hill; 1994.

[36] Blair CE, Glaister J, Brown A, Phillips C (2007). Fostering activities of daily living by intact nursing home residents. Educ Gerontol.

[37] Grol R, Wensing M (2004). What drives change? Barriers to and incentives for achieving evidence-based practice. Med J Aust.

[38] Benjamin K, Edwards N, Ploeg J, Legault F (2014). Barriers to physical activity and restorative care for residents in long-term care: a review of the literature. J Aging Phys Act.

[39] McDowell N, McKenna J, Naylor P (1997). Factors that influence practice nurses to promote physical activity. Br J Sports Med.

[40] Huijg JM, Gebhardt WA, Verheijden MW, van der Zouwe N, de Vries JD, Middelkoop BJ, Crone MR (2015). Factors influencing primary health care professionals’ physical activity promotion behaviors: a systematic review. Int J Behav Med.

[41] Ribera AP, McKenna J, Riddoch C (2005). Attitudes and practices of physicians and nurses regarding physical activity promotion in the Catalan primary health-care system. Eur J Public Health.

[42] den Ouden M, Bleijlevens MH, Meijers JM, Zwakhalen SM, Braun SM, Tan FE, Hamers JP (2015). Daily (in) activities of nursing home residents in their wards: an observation study. J Am Med Dir Assoc.

