# Supplementary material for: The perceived behavior and barriers of community care professionals in encouraging functional activities of older adults: the development and validation of the MAINtAIN-C questionnaire
Source: BMC Health Serv Res. 2020 Sep 29;20:907. doi: 10.1186/s12913-020-05762-w (PMC7526165; doi:10.1186/s12913-020-05762-w)
Supplement: Supplementary file 1 — Additional File: 1 Supplementary Table 1 and Supplementary Table 2. show the complete factor loadings on the MAINtAIN-C Behaviors and Barriers scales. [file 12913_2020_5762_MOESM1_ESM.pdf]

Supplementary Table 1 Factor loadings after Oblimin rotation in the EFA of the MAINtAIN-C Behaviors scale ($N = 79$)

Items	Four factors				Three factors			Two factors	
	**1**	**2**	**3**	**4**	**1**	**2**	**3**	**1**	**2**
1	.246	-.118	**-.360**	.084	.288	-.127	**-.353**	**.566**	-.090
2	.133	.023	**-.743**	.131	.249	-.003	**-.715**	**.825**	.056
3	-.200	-.152	**-.834**	.082	-.016	-.195	**-.704**	**.579**	-.135
4	.061	-.210	**-.377**	.359	**.373**	-.231	-.276	**.575**	-.200
5	-.050	**-.749**	.095	.255	.156	**-.755**	.168	-.001	**-.763**
6	**.498**	.011	-.247	.313	**.689**	.015	-.221	**.796**	.034
7	.053	.018	.031	**.621**	**.515**	.003	.120	**.342**	-.011
8	.103	-.214	**-.419**	.369	**.415**	-.236	-.320	**.650**	-.202
9	**.369**	-.297	-.005	.139	**.412**	-.294	-.006	**.371**	-.290
10	**.445**	.123	-.271	.117	**.476**	.124	-.284	**.674**	.157
11	**.547**	-.008	-.206	.288	**.711**	-.002	-.185	**.780**	.012
12	-.192	**-.857**	-.084	.083	-.097	**-.874**	-.021	-.064	**-.872**
13	-.191	**-.958**	-.062	-.044	-.220	**-.985**	-.031	-.159	**-.975**
14	.082	**-.770**	-.127	.006	.052	**-.791**	-.117	.151	**-.779**
15	.182	**-.786**	-.147	-.101	.039	**-.801**	-.174	.191	**-.781**
16	.119	**-.767**	.075	-.057	.015	**-.776**	.055	-.032	**-.781**
17	.400	**-.545**	.003	-.071	.232	**-.540**	-.060	.262	**-.531**
18	.420	**-.422**	-.156	-.163	.191	**-.420**	-.224	.369	**-.395**
19	**.386**	-.322	-.006	.297	**.561**	-.320	.027	**.469**	-.318
20	.099	.049	**-.664**	-.210	-.068	.025	**-.699**	**.520**	.073

Factor Correlations

Factors	1	2	3	4	1	2	3	1	2
**1**	1	-.33	-.47	.30	1	-.43	-.46	1	-.495
**2**	-.33	1	.37	-.28	-.43	1	.34	-.495	1
**3**	-.47	.37	1	-.27	-.46	.34	1		
**4**	.30	-.28	-.27	1					

*The EFA was conducted using principal axis factoring and a direct Oblimin (oblique) rotation; factor loadings in boldface are the highest loading of that item.

Supplementary Table 2 Factor loadings after Oblimin rotation in the EFA of the MAINtAIN-C Barriers scale (*N* = 79)

**Items**	**Four factors**				**Three factors**		
Items concerning barriers related to the context of the clients							
1	.072	-.059	.263	**.409**	.176	-.189	**.290**
2	.006	**.475**	-.044	-.247	-.010	**.553**	-.079
3	.218	-.124	-.062	**.287**	**.289**	-.228	-.030
4	**.359**	-.023	-.136	.235	**.427**	-.115	-.108
5	-.116	.360	.191	**-.387**	-.195	**.496**	.144
6	.204	**.606**	-.021	-.281	.189	**.695**	-.058
7	.023	**.685**	-.061	.231	.178	**.538**	-.058
8	-.014	**.705**	-.072	.036	.090	**.651**	-.091
9	.070	**.604**	.028	.034	.154	**.567**	.013
10	-.027	**.475**	.283	-.323	-.082	**.592**	.242
Items concerning barriers related to the professional context							
11	-.048	-.121	**.512**	.032	-.082	-.099	**.525**
12	.102	.089	**.487**	.072	.106	.089	**.499**
13	-.140	.009	**.532**	.182	-.107	-.029	**.543**
14	**.476**	-.072	-.091	.014	**.467**	-.074	-.077
15	**.573**	-.015	.055	.010	**.559**	-.006	.073
16	-.085	.065	**.382**	-.087	-.126	.120	**.377**
17	**.783**	.133	-.103	-.141	**.733**	.187	-.092
18	.064	.050	**.445**	.325	.149	-.046	**.463**
19	.063	.169	.151	**.379**	**.191**	.027	.173
20	-.042	-.050	-.004	**.258**	.034	**-.147**	.016
Items concerning barriers related to the social context: team functioning							
21	**.459**	.127	.114	.238	**.532**	.045	.141
22	**.810**	.227	-.123	.034	**.842**	.212	-.111
23	**.298**	.124	.169	.208	**.363**	.054	.189
24	.145	-.264	**.305**	-.141	.049	-.177	**.303**
25	**.657**	-.023	-.081	-.039	**.629**	-.001	-.065
26	**.306**	.274	.224	.203	**.384**	.204	.237
Items concerning barriers related to the organizational context							
27	.430	-.036	**.483**	-.191	.330	.073	**.467**
28	**.397**	.092	.141	.082	**.418**	.073	.156
29	**.373**	-.026	.135	-.132	**.310**	.039	.136
30	**.468**	-.239	.189	.001	**.416**	-.211	.210
31	.101	.000	**.227**	-.066	.067	.040	**.224**
32	**.519**	.050	.140	.041	**.521**	.049	.155
33	.162	-.023	**.499**	.078	.153	-.020	**.517**
	**Factor-Factor correlations**						
**Factors**	**1**	**2**	**3**	**4**	**1**	**2**	**3**
**1**	1	.07	.28	.19	1	-.02	.30
**2**	.07	1	.07	-.11	-.02	1	.04
**3**	.28	.07	1	.04	.30	.04	1
**4**	.19	-.11	.04	1			

*The EFA was conducted using principal axis factoring and a direct Oblimin (oblique) rotation; factor loadings are bolded as they represent the items that most contribute towards the respective factors.
