# Supplementary material for: The perceived behavior and barriers of community care professionals in encouraging functional activities of older adults: the development and validation of the MAINtAIN-C questionnaire
Source: BMC Health Serv Res. 2020 Sep 29;20:907. doi: 10.1186/s12913-020-05762-w (PMC7526165; doi:10.1186/s12913-020-05762-w)
Supplement: Supplementary file 2 — Additional File 2. The MAINtAIN-C questionnaire. The MAINtAIN-C shows the MAINtAIN-C Behaviors scale and MAINtAIN-C Barriers scale [file 12913_2020_5762_MOESM2_ESM.pdf]

MAINtAIN-C questionnaire

The MAINtAIN-C questionnaire consists of the MAINtAIN-C Behaviors scale to measure the perceived behavior in encouraging functional activities (20 items) and the MAINtAIN-C Barriers scale to measure the perceived barriers in encouraging functional activities related to the clients' context (7 items) and the professional, social, and organizational contexts (21 items), of community care professionals. To ensure transparency and comparability in the interpretation and analysis of the questionnaire, a description is provided on how to interpret the results and how the missing values can be handled.

Interpretation of the MAINtAIN-C Behaviors

The sum score on the total scale, for each respondent varying from 20 to 180, indicates the degree to which the respondent perceives to encourage functional activities.

Interpretation of the MAINtAIN-C Barriers

The scores of the positively formulated items should be reversed so that higher scores always indicate stronger experienced barriers. Furthermore, the sum score per subscale—varying from 7 to 63 for the clients' context and from 21 to 189 for the professional, social, and organizational contexts, for each respondent—indicates the degree to which the respondent is perceived to experience barriers in encouraging functional activities.

Handling the missing values of the MAINtAIN-C

To ensure comparability and transparency in scores, the average score of all respondents on all items in that scale can be imputed, if at least 80% of the items of that scale had been completed by the respondent.

MAINtAIN-C Behaviors

1.	I closely follow the extent to which clients are able to perform their own ADLs.	Never ○	○	○	○	Sometimes ○	○	○	○	Always ○
2.	I encourage clients to perform their own ADLs as much as possible.	Never ○	○	○	○	Sometimes ○	○	○	○	Always ○
3.	I closely follow the extent to which assistive devices are available and adequately used by clients.	Never ○	○	○	○	Sometimes ○	○	○	○	Always ○
4.	I advise clients about the added value of consulting other disciplines (for example physical therapy) in order to encourage the independent performance of ADLs.	Never ○	○	○	○	Sometimes ○	○	○	○	Always ○
5.	I discuss with new clients if a meal can be eaten independently.	Never ○	○	○	○	Sometimes ○	○	○	○	Always ○
6.	I encourage clients to independently dress and undress.	Never ○	○	○	○	Sometimes ○	○	○	○	Always ○
7.	I complement clients when they dress and undress themselves.	Never ○	○	○	○	Sometimes ○	○	○	○	Always ○
8.	I closely follow the extent to which clients are to move about within their home.	Never ○	○	○	○	Sometimes ○	○	○	○	Always ○
9.	I encourage clients to participate in activities outside their home.	Never ○	○	○	○	Sometimes ○	○	○	○	Always ○
10.	I encourage clients to independently wash and dry themselves.	Never ○	○	○	○	Sometimes ○	○	○	○	Always ○
11.	I encourage clients to use the toilet independently.	Never ○	○	○	○	Sometimes ○	○	○	○	Always ○

12.	I discuss with clients if they can prepare their meals independently.	Never ○	○	○	○	Sometimes ○	○	○	○	Always ○
13.	I discuss with clients if they can do their shopping independently.	Never ○	○	○	○	Sometimes ○	○	○	○	Always ○
14.	I discuss with clients if they can wash their clothes independently.	Never ○	○	○	○	Sometimes ○	○	○	○	Always ○
15.	I discuss with clients if they can do ‘light housework’ independently, for example washing/drying dishes.	Never ○	○	○	○	Sometimes ○	○	○	○	Always ○
16.	I discuss with clients if they can mop the bathroom independently after getting washed.	Never ○	○	○	○	Sometimes ○	○	○	○	Always ○
17.	I discuss with clients which activities they used to do and they still can perform (ADLs, IADLs, social activities).	Never ○	○	○	○	Sometimes ○	○	○	○	Always ○
18.	I discuss with clients which activities (ADLs, IADLs, social activities) they would really like to perform themselves.	Never ○	○	○	○	Sometimes ○	○	○	○	Always ○
19.	I encourage the clients’ family and informal caregivers to promote self-reliance in clients.	Never ○	○	○	○	Sometimes ○	○	○	○	Always ○
20.	If ‘encourage self-reliance and independence’ is included in the care plan, then I follow this.	Never ○	○	○	○	Sometimes ○	○	○	○	Always ○

MAINtAIN-C Barriers

Context of the clients

**1.**	Clients are often able to perform ADLs more independently than they now do.	Completely disagree ◯	◯	◯	◯	Neither agree, nor disagree ◯	◯	◯	◯	Completely agree ◯
**2.**	Clients are afraid to walk on their own, without help from others.	Never ◯	◯	◯	◯	Sometimes ◯	◯	◯	◯	Always ◯
**3.**	Clients ask for help with ADLs so that they can get extra attention.	Never ◯	◯	◯	◯	Sometimes ◯	◯	◯	◯	Always ◯
**4.**	Family or informal caregivers expect the nurses and nurse assistants to take over the activities that clients themselves can still perform.	Never ◯	◯	◯	◯	Sometimes ◯	◯	◯	◯	Always ◯
**5.**	Clients do not want to perform activities themselves such as bathing or opening/closing curtains even if they still can.	Never ◯	◯	◯	◯	Sometimes ◯	◯	◯	◯	Always ◯
**6.**	Financial limitations restrict clients in performing ADLs and IADLs independently.	Never ◯	◯	◯	◯	Sometimes ◯	◯	◯	◯	Always ◯
**7.**	An overburdened family or informal caregiver limits clients in performing ADLs and IADLs independently	Never ◯	◯	◯	◯	Sometimes ◯	◯	◯	◯	Always ◯

Context of the professionals, the social context (the team functioning) and the organizational context.

**1.**	I think that organizing my work so that clients are ready on time is more important than clients performing ADLs independently.	Never ○	○	○	○	Sometimes ○	○	○	○	Always ○
**2.**	I am afraid that clients will hurt themselves if I encourage them to walk alone.	Never ○	○	○	○	Sometimes ○	○	○	○	Always ○
**3.**	It is primarily the responsibility of the physical therapist or occupational therapist to encourage clients to perform activities.	Completely disagree ○	○	○	○	Neither agree, nor disagree ○	○	○	○	Completely agree ○
**4.**	Within my team, we think that it is important to encourage clients to perform ADLs as much as possible independently.	Completely disagree ○	○	○	○	Neither agree, nor disagree ○	○	○	○	Completely agree ○
**5.**	Within my team, we think that it is our task to inform the family or informal caregivers about the importance of clients performing activities independently.	Completely disagree ○	○	○	○	Neither agree, nor disagree ○	○	○	○	Completely agree ○
**6.**	Within my team, sufficient expertise is available to encourage clients to be as independent as possible in performing ADLs (such as bathing, moving about).	Completely disagree ○	○	○	○	Neither agree, nor disagree ○	○	○	○	Completely agree ○
**7.**	Encouraging independence as much as possible in clients' ADLs, IADLs and social activities gives me less time for other things.	Completely disagree ○	○	○	○	Neither agree, nor disagree ○	○	○	○	Completely agree ○
**8.**	I find it difficult to encourage clients to be self-reliant and independent.	Completely disagree ○	○	○	○	Neither agree, nor disagree ○	○	○	○	Completely agree ○

9.	Within my team, the collaboration with experts (for example occupational or physical therapists) is not good enough to encourage clients in performing ADLs as independently as possible.	Completely disagree<br>○	○	○	○	Neither agree, nor disagree<br>○	○	○	○	Completely agree<br>○
10.	I can count on enough support from my colleagues when I allow clients to perform ADLs and IADLs as independently as possible.	Completely disagree<br>○	○	○	○	Neither agree, nor disagree<br>○	○	○	○	Completely agree<br>○
11.	The manager of my team considers it important that clients perform ADLs and IADLs as independently as possible.	Never<br>○	○	○	○	Sometimes<br>○	○	○	○	Always<br>○
12.	I speak to my colleagues when I hear that they perform activities that clients can still perform themselves.	Never<br>○	○	○	○	Sometimes<br>○	○	○	○	Always<br>○
13.	The team discusses how we can encourage clients to perform ADLs and IADLs as independently as possible.	Never<br>○	○	○	○	Sometimes<br>○	○	○	○	Always<br>○
14.	Within my team, it is our routine to take over the ADLs and IADLs (such as making sandwiches) for our clients.	Never<br>○	○	○	○	Sometimes<br>○	○	○	○	Always<br>○

**15.**	My organization is not geared towards involving clients in the performance of ADLs and IADLs (such as independently bathing and dressing or preparing a meal).	Completely disagree ○ ○ ○ ○ Neither agree, nor disagree ○ ○ ○ ○ Completely agree ○
**16.**	In my organization, there are enough people available who know how to encourage self-reliance and independent performance of activities by clients.	Completely disagree ○ ○ ○ ○ Neither agree, nor disagree ○ ○ ○ ○ Completely agree ○
**17.**	My organization offers the possibility to follow internal or external courses that focus on encouraging clients' physical activity	Completely disagree ○ ○ ○ ○ Neither agree, nor disagree ○ ○ ○ ○ Completely agree ○
**18.**	In my organization, we do not have agreements or guidelines concerning how we can encourage clients' physical activity.	Completely disagree ○ ○ ○ ○ Neither agree, nor disagree ○ ○ ○ ○ Completely agree ○
**19.**	I have inadequate time to activate clients to be self-reliant because of the-needs assessment determined by the community nurse in my team.	Completely disagree ○ ○ ○ ○ Neither agree, nor disagree ○ ○ ○ ○ Completely agree ○
**20.**	Encouraging self-reliance and independence has a high priority in my organization.	Completely disagree ○ ○ ○ ○ Neither agree, nor disagree ○ ○ ○ ○ Completely agree ○
**21.**	There is a structural shortage of staff available to encourage clients to perform ADLs and IADLs (such as independently bathing and dressing or preparing a meal) as independently as possible.	Completely disagree ○ ○ ○ ○ Neither agree, nor disagree ○ ○ ○ ○ Completely agree ○
